# Climate Change Impact on the Potential Distribution and Invasion Risk of *Tetramorium caldarium* (Roger, 1857): A Species Distribution Modeling Approach Using MaxEnt

**DOI:** 10.1002/ece3.74319

**Published:** 2026-09-13

**Authors:** Mohamed Nasser, Mostafa R. Sharaf, Areej A. AlKhalaf, Francisco Hita‐Garcia, Alexander Radchenko, Stephen Judd, Mariam S. Alqaydi, Meera M. Alteneiji, Sara AlAshaal

**Affiliations:** ^1^ Research Lab of Biogeography and Wildlife Parasitology, Department of Entomology, Faculty of Science Ain Shams University Cairo Egypt; ^2^ Al Dhaid Wildlife Centre Environment and Protected Areas Authority Sharjah UAE; ^3^ Biology Department, College of Science Princess Nourah Bint Abdurrahman University Riyadh Saudi Arabia; ^4^ Center for Integrative Biodiversity Discovery Museum für Naturkunde Berlin Germany; ^5^ I.I. Schmalhausen Institute of Zoology of National Academy of Sciences of Ukraine Kiev Ukraine; ^6^ Zoology Department National Museums Liverpool Liverpool UK

**Keywords:** biosecurity, climate change, introduced species, MaxEnt, niche modeling, species distribution modeling, tramp ants

## Abstract

Climate change is reshaping global species distributions and altering invasion dynamics, yet our understanding of how thermophilic invasive species will respond to future climatic conditions remains incomplete. We employed Maximum Entropy (MaxEnt) species distribution modeling to evaluate current and future suitable habitat for *Tetramorium caldarium* (Roger, 1857), a pantropical tramp ant species with established exotic populations across six continents. Three hundred and forty‐six filtered occurrence records and five bioclimatic variables (bio_1, bio_4, bio_7, bio_11, bio_12) were selected through correlation analysis. Models were projected onto future climate surfaces using MRI‐CGCM3 outputs for two Representative Concentration Pathways (RCP 2.6 and RCP 8.5) at 2050 and 2070. The model demonstrated excellent predictive performance (AUC = 0.942, TSS = 0.68), revealing that *T. caldarium* currently occupies extensive suitable habitat across tropical and subtropical regions between 30° N and 30° S. Three‐dimensional niche analysis demonstrated remarkable climatic breadth, with the species occurring across temperature gradients from 14°C to 29°C and precipitation regimes from 500 to 3500+ mm annually. Jackknife analysis revealed that temperature seasonality (43.7%) and coldest quarter temperature (25.9%) were primary limiting factors, while precipitation contributed minimally (4.5%). Future projections indicated relatively stable core distributions through 2070, with net habitat gains ranging from 275,000 km^2^ (RCP 2.6, 2050) to 630,000 km^2^ (RCP 8.5, 2070). Southern China, southeastern United States, Mediterranean Basin, and northern Argentina were identified as high‐risk regions for future spread. *T. caldarium* possesses sufficient climatic niche breadth to maintain its spread potential despite climate change, with warming temperatures creating new opportunities in temperate regions. These findings emphasize that preventing human‐mediated dispersal remains more critical than managing climate‐driven range shifts for this globally significant invasive species.

## Graphical abstract

We employed maximum entropy species distribution modeling to assess current and future suitable habitat for *Tetramorium caldarium* , a pantropical invasive ant species, using 346 occurrence records and bioclimatic variables projected under two climate scenarios (RCP 2.6 and RCP 8.5) for 2050 and 2070. The model demonstrated excellent performance (AUC = 0.942, TSS = 0.68) and revealed that *T. caldarium* possesses exceptional climatic breadth, with temperature seasonality and cold‐season temperatures serving as primary limiting factors rather than precipitation. Future projections indicate relatively stable core distributions through 2070 with net habitat gains of 2.3%–4.5%, identifying southern China, southeastern United States, Mediterranean Basin, and northern Argentina as high‐risk regions for future invasion, emphasizing that preventing human‐mediated dispersal remains more critical than managing climate‐driven range shifts for this globally significant species.

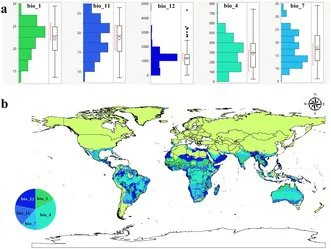

## 1 Introduction

Climate change is the primary modern trigger for species redistribution and biological invasions worldwide. Increasing temperatures, modified precipitation patterns, and extreme weather phenomena are profoundly transforming the biological landscape, facilitating the establishment and expansion of invasive species (Hellmann et al. 2008; Walther et al. 2009; Bellard et al. 2013). Ants have emerged as sensitive indicators of environmental changes due to their ectothermic nature and significant reliance on climatic variables for survival and reproduction (Dunn et al. 2009; Jenkins et al. 2011; Diamond et al. 2012). The reaction of ants to climate change is intricate and non‐linear; mild temperature rises may enhance colony sizes and species variety, whereas excessive warming could yield adverse consequences (Kaspari et al. 2019; Bishop et al. 2022). Comprehending the impact of climate change on the distribution patterns of invasive ant species is essential for formulating efficient management methods and averting ecological disturbances in susceptible habitats (Angulo et al. 2022; Liu et al. 2025).

“Tramp” ants are a small percentage of transported ants that are widely distributed geographically and have strong ties to cities and human activity (Passera 1994). Even though tramp ants are frequently found in areas with human populations, native ants may not always face competition from them (McGlynn 1999). Native ants are outcompeted by invasive ants that invade natural regions and are either disturbed or left undisturbed (Wetterer and Hita Garcia 2015). *Tetramorium caldarium* (Roger, 1857) illustrates the intricate interplay between climatic variations and biological introductions. This tramp species, originally indigenous to Africa, has attained extensive global distribution via human trade, establishing colonies in tropical and subtropical locations worldwide (Wetterer and Hita Garcia 2015; McGlynn 1999). The species has significant ecological plasticity and adaptability, traits that have enabled its successful colonization of several habitats, including urban settings and natural ecosystems (Bolton 1995; Fisher and Cover 2007). Recent studies indicate that *T. caldarium* typically demonstrate smaller climatic niche shifts than native species, implying that their success may stem from pre‐adaptation to varied climatic conditions rather than extraordinary niche plasticity (Bertelsmeier et al. 2020). This adaptability raises considerable concerns over its possible reaction to shifting climatic conditions and the ensuing ramifications for spread dynamics in previously inhospitable areas. The biology of tramp species and their potential ecological impact on populations of native ant species in the ecosystems they invade are poorly understood. While populations in tropical and subtropical locations are confined to greenhouses, zoos, and heated buildings (Bolton 1980), *T. caldarium* is typically found in disturbed habitats with increased anthropogenic activity (Bharti and Kumar 2012).

The prevalence capacity of *T. caldarium* transcends its existing distribution limits, especially as climate change persistently modifies global environmental circumstances. The species demonstrates an extensive latitudinal distribution from tropical to temperate regions, indicating significant climate adaptability that could facilitate range expansion in warmer conditions (Schlick‐Steiner et al. 2006; Seifert 2003). Historical records demonstrate that *T. caldarium* has successfully formed populations in areas with distinct climatic circumstances, ranging from maritime islands to continental landmasses, illustrating its ability to exploit various ecological niches (Wagner et al. 2017; Ward 2005). Recent extensive assessments of foreign ant species indicate that more than 500 species have been disseminated globally by human activities, with numerous species demonstrating potential for continued proliferation in response to shifting environmental conditions (Bertelsmeier et al. 2021). The species' ecological adaptability, coupled with persistent climate change, necessitates an urgent evaluation of its future spread potential invasion concerns (Lee et al. 2021).

Species distribution modeling (SDM) has become an effective instrument for forecasting the probable geographic ranges of invasive and tramp species under present and future climatic conditions (Nasser, Sharaf, Wetterer, et al. 2025b). Among the several SDM methodologies, Maximum Entropy (MaxEnt) modeling has garnered significant acclaim for its effective performance in forecasting species distributions, especially using presence‐only occurrence data (Phillips et al. 2006; Phillips and Dudík 2008). MaxEnt has exhibited exceptional efficacy in predicting the distributions of both invasive and tramp species (Merow et al. 2013; Elith et al. 2011). The method's capacity to manage intricate environmental interactions and yield probabilistic results renders it especially significant for invasion biology, where comprehending establishment probabilities is essential for management strategies (Jiménez‐Valverde et al. 2011; West et al. 2016). Recent advancements in MaxEnt parameterization and ensemble modeling techniques have improved the accuracy of distribution forecasts for invasive and tramp species (Morales et al. 2017; Kim et al. 2022).

The amalgamation of climate change forecasts with species distribution models establishes a framework for predicting future spread and invasion scenarios and pinpointing areas at heightened danger. Recent research on invasive and tramp species has demonstrated varied reactions to climate change, with certain species undergoing range contractions while others extend their territories in future climatic scenarios (Fitzpatrick et al. 2007; Roura‐Pascual et al. 2011). Modeling studies indicate that *Solenopsis invicta* Buren, 1972 may experience range extension due to climate change, whereas the distribution of *Anoplolepis gracilipes* (Smith, 1857) is likely to stay rather steady (Morrison et al. 2004; Kim et al. 2022). The diverse reactions emphasize the species‐specific effects of climate change and underline the necessity for thorough evaluations of individual invasive and tramp taxa (Early et al. 2016). The extensive ecological tolerance of *T. caldarium* , coupled with continuous global warming, may facilitate its range extension into formerly climatically unsuitable places, especially in temperate countries undergoing warming trends (Guénard et al. 2012).

Invasive ants affect native ecosystems by means of direct predation, competition with indigenous species, and modification of ecological processes, including seed dispersal and soil dynamics (Holway et al. 2002; Lach et al. 2010). Moreover, the species' propensity to form high‐density colonies in both natural and human‐altered ecosystems can result in significant economic repercussions due to damage to infrastructure, agricultural systems, and public health issues (Pimentel et al. 2005; Bradshaw et al. 2016). Recent economic evaluations of the effects of invading ants have projected annual damages in the billions globally, underscoring the necessity for proactive management strategies (Diagne et al. 2021). *Tetramorium caldarium* is currently considered to be a cosmopolitan tramp ant that is exotic in most regions where it is found and shows no evidence of strong invasive potential. However, if this changes, then possible repercussions of *T. caldarium* range expansion extend well beyond mere geographic redistribution and may involve substantial ecological, economic, and societal ramifications in the future. Comprehending the prospective dissemination potential of *T. caldarium* is crucial for formulating proactive management strategies and allocating resources for preventative and control initiatives (Hulme 2006; Blackburn et al. 2011).

This study seeks to examine the present and prospective suitable habitat for *T. caldarium* by MaxEnt species distribution modeling, offering thorough evaluations of potential distribution across diverse climate change scenarios. By incorporating comprehensive occurrence data with bioclimatic characteristics and future climate forecasts, we aim to pinpoint areas most susceptible to spread and assess the probable extent of range extension. The results will enhance our comprehension of climate‐induced biological distribution and offer significant insights for adaptive management tactics in the face of global environmental change. We seek to improve our ability to forecast the global potential distribution of tramp species in a swiftly evolving environment.

## 2 Materials and Methods

### 2.1 Occurrence Data Collection and Preparation

**FIGURE 1:**
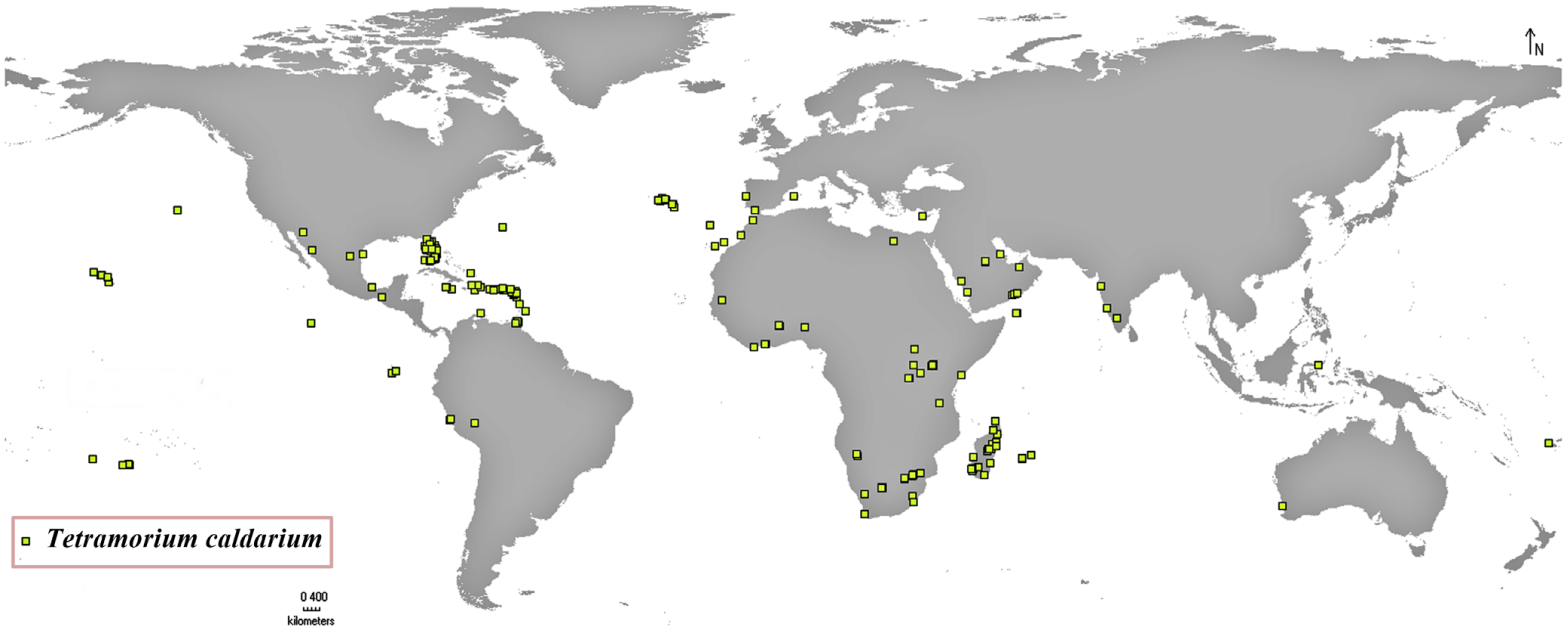
The final set of occurrence points of *Tetramorium caldarium* that was used in this study.

On June 15, 2025, *Tetramorium caldarium* species occurrence records were retrieved from the Global Biodiversity Information Facility (GBIF) database (GBIF.org 2025). To guarantee data quality and lessen spatial bias, the original dataset was put through a rigorous filtering process. Records with the same geographic coordinates were eliminated, and events with coordinate uncertainty more than 10 km or without accurate geospatial data were not included in the analysis (Hijmans 2012). We manually identified and eliminated records that showed clear geographic inaccuracies, such as those falling in maritime habitats or showing improbable coordinate‐locality mismatches. We used a spatial thinning protocol with a minimum distance threshold of 10 km between occurrence points to minimize sampling bias and spatial autocorrelation. This helps minimize the impact of collection bias while preserving a sufficient sample size for model training (Boria et al. 2014; Aiello‐Lammens et al. 2015). A final dataset of 346 occurrence records was kept for SDM after these filtering processes (Figure 1). Given that prior research has shown dependable model performance with comparable or smaller sample sizes when suitable model settings are used, this sample size is deemed sufficient for MaxEnt modeling (Wisz et al. 2008; van Proosdij et al. 2016).

### 2.2 Environmental Variables and Data Processing

The WorldClim version 2.1 database, which represents average climatic conditions for the years 1970–2000, provided the current bioclimatic variables at a spatial resolution of 2.5 arc‐minutes (~5 km at the equator) (Fick and Hijmans 2017). The WorldClim bioclimatic variables are biologically meaningful summaries of temperature and precipitation patterns that are frequently utilized in SDM applications. They are created from monthly temperature and precipitation data (Booth et al. 2014). Since multicollinearity can result in overfitting and impair model interpretability, we first performed a thorough correlation analysis to identify and eliminate highly collinear variables (|*r*| > 0.7) among the 19 bioclimatic variables that were available (Dormann et al. 2013). Using JMP Pro 16 statistical software (SAS Institute Inc., Cary, NC, USA), correlation analysis was carried out to look at pairwise Pearson correlation coefficients between all bioclimatic variables in the research area. Five variables—Bio1 (Annual Mean Temperature), Bio4 (Temperature Seasonality, coefficient of variation), Bio7 (Temperature Annual Range), Bio11 (Mean Temperature of Coldest Quarter), and Bio12 (Annual Precipitation)—were chosen for the final model construction based on correlation structure and ecological relevance to ant biogeography. Together, these factors reflect the basic climatic factors that impact the distribution of ectothermic species, especially temperature and seasonality (Kaspari et al. 2015; Angilletta et al. 2002).

We used the outputs of the MRI‐CGCM3 (Meteorological Research Institute Coupled General Circulation Model version 3) global climate model over two future time periods (2050 and 2070) under two Representative Concentration Pathways (RCP 2.6 and RCP 8.5) in order to project future climates (Yukimoto et al. 2012). While RCP 8.5 represents a high‐emission, business‐as‐usual scenario with radiative forcing reaching 8.5 W/m^2^ by 2100, RCP 2.6 represents a low‐emission scenario assuming significant mitigation efforts, with radiative forcing peaking at about 3 W/m^2^ before 2100 and then declining (van Vuuren et al. 2011; Riahi et al. 2011). Based on its proven capacity to accurately replicate tropical and subtropical climate trends and its accessibility via the WorldClim database at the right spatial resolution for modeling the distribution of species on a global scale, the MRI‐CGCM3 model was chosen (McSweeney et al. 2015). ArcGIS 10.8 (Environmental Systems Research Institute, Redlands, CA, USA) was used to process all climate data layers, both present and future, in order to guarantee uniform spatial extent, resolution, and projection. To maintain consistency and computational efficiency across modeling scenarios, all raster layers were trimmed to the same geographic area and converted to ASCII format (.asc) as needed for MaxEnt software input.

### 2.3 Modeling Species Distribution

MaxEnt version 3.4.4, a machine learning algorithm founded on the principle of MaxEnt, was used to model the distribution of species. It has proven to be a reliable tool for modeling species distributions with presence‐only occurrence data (Phillips et al. 2006; Phillips and Dudík 2008; Elith et al. 2011). By determining the distribution of MaxEnt (most dispersed or closest to uniform) at known presence locales, MaxEnt calculates the probability distribution of species occurrence, subject to limitations imposed by environmental parameters (Phillips et al. 2017). The program determines environmental parameters that best differentiate suitable from unsuitable habitat by comparing background environmental conditions throughout the research region with environmental conditions at presence locales (Nasser, Sharaf, Aldawood, et al. 2025a).

Recent best‐practice guidelines for MaxEnt applications were adhered to when parameterizing the model (Merow et al. 2013; Morales et al. 2017). Since these settings have been demonstrated to offer the best balance between model complexity and predictive performance across a variety of taxa and sample sizes, we used the default regularization multiplier (*β* = 1) and auto‐features settings, which automatically choose the appropriate feature classes (linear, quadratic, product, threshold, and hinge) based on sample size (Radosavljevic and Anderson 2014; Phillips and Dudík 2008). 75% of the occurrence records were used to train the model, while the remaining 25% were set aside for model evaluation using random *k*‐fold cross‐validation with 10 replicates. While taking into consideration the uncertainty brought on by random data partitioning, this cross‐validation method yields reliable estimates of model performance (Fielding and Bell 1997; Muscarella et al. 2014). To guarantee proper model training, the model convergence criterion was set at 10^−5^ with a maximum of 5000 iterations. Estimates of habitat suitability ranged from 0 (lowest suitability) to 1 (best suitability), with the output format set to logistic probability (Phillips and Dudík 2008).

At a sample size of 10,000 points, which is frequently used to provide sufficient characterization of background environmental conditions while maintaining computational efficiency, background points (pseudo‐absences) were randomly selected from the study area (Barbet‐Massin et al. 2012; Phillips and Dudík 2008). The worldwide terrestrial surface was chosen as the study area because it reflects the species' capacity for long‐distance establishment and spread across a variety of biogeographic zones through human activity. Using the same model parameters and variable changes as current distribution modeling, models calibrated on current climate data were projected onto future climatic surfaces for every RCP scenario and time period combination (Elith et al. 2010).

### 2.4 Analysis and Evaluation of the Model

Several complementing measures that measure various facets of predictive accuracy were used to evaluate the model's performance. The model's capacity to distinguish between presence and background locales was measured using the area under the receiver operating characteristic curve (AUC); AUC values > 0.9 indicate strong discriminating abilities (Swets 1988; Fielding and Bell 1997). Additionally, we computed the true skill statistic (TSS), which is independent of prevalence and takes into account both omission and commission mistakes. TSS values > 0.6 are thought to be a sign of high model performance (Allouche et al. 2006; Liu et al. 2013). To get reliable performance estimates, both measures were averaged throughout the ten cross‐validation repeats.

Jackknife tests, which measure the improvement in model performance when each variable is utilized separately and when each variable is removed from the entire model, were used to evaluate the significance of each variable in MaxEnt (Phillips et al. 2017). This method finds factors that, when used alone, most restrict the distribution of species and variables that provide special information not found by other predictors. To depict the relationship between habitat appropriateness and specific bioclimatic factors, response curves were created for each environmental variable. This gave information about the species' environmental tolerances and ideal circumstances (Elith et al. 2011).

Using DIVA‐GIS version 7.5 software (Hijmans et al. 2001), limiting factor analysis was performed to determine which environmental characteristic most restricts species distribution at each point within the study region. Insight into the regional patterns of environmental limitations on species distribution is provided by this research, which generates maps displaying the spatial distribution of limiting factors and quantifies the relative area affected by each variable (Ramirez‐Villegas et al. 2010). The species' climatic niche in multidimensional environmental space defined by Bio4, Bio7, and Bio11 was examined using three‐dimensional niche visualization using JMP Pro 16 software. This showed the range of environmental conditions occupied by the species as well as possible areas of niche overlap or divergence.

We performed binary classification of continuous suitability outputs using the maximum training sensitivity plus specificity threshold, which has been demonstrated to offer the best trade‐off between sensitivity and specificity for presence‐only models (Liu et al. 2013; Jiménez‐Valverde and Lobo 2007), in order to quantify changes in habitat suitability under future climate scenarios. To identify regions of habitat loss (areas suitable under current climate but unsuitable under future climate), habitat gain (areas unsuitable currently but suitable in future), and stable habitat (areas maintaining suitability status), areas were categorized as suitable or unsuitable based on this threshold. Future distributions were then compared with current distributions. To determine the size of anticipated range shifts, the amount of suitable habitat was measured for each scenario, and percentage changes were computed.

## 3 Results

### 3.1 Variable Correlation Analysis

**FIGURE 2:**
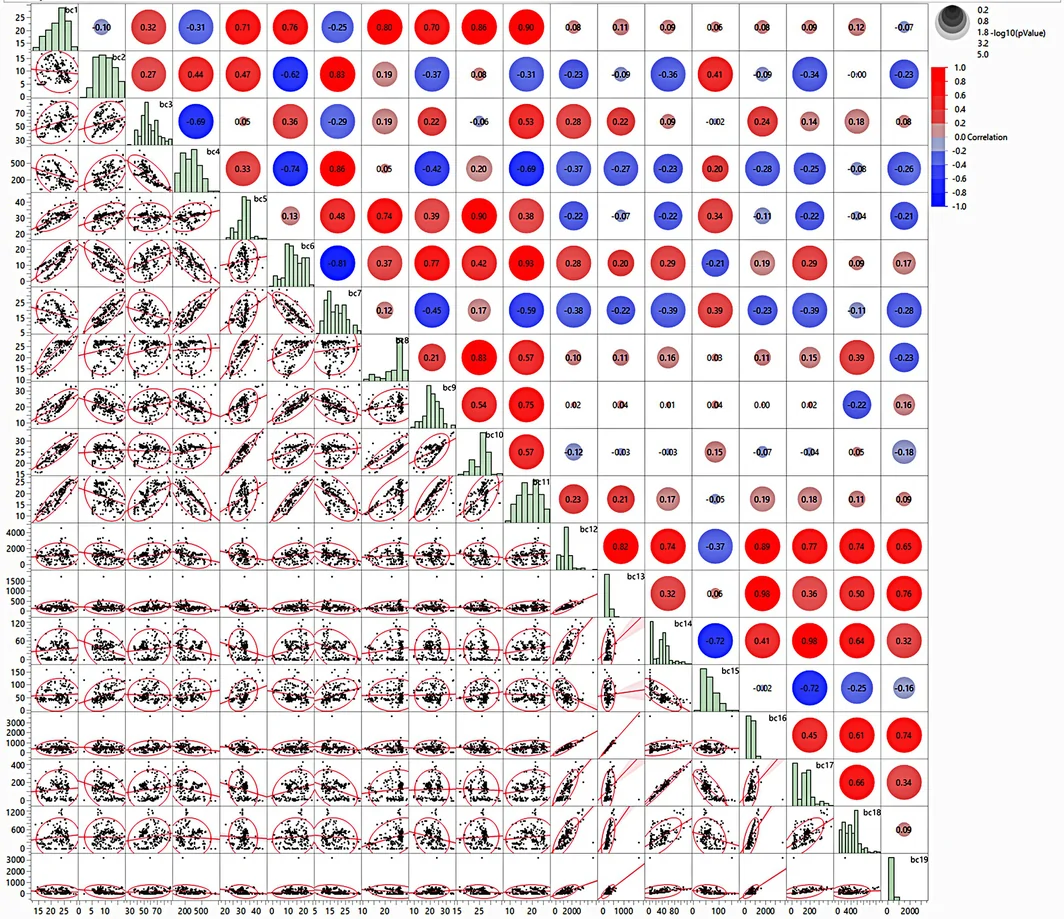
Correlation matrix and pairwise relationships among bioclimatic variables considered for species distribution modeling of *Tetramorium caldarium* . The figure displays a comprehensive correlation analysis of 19 bioclimatic variables (bio1–bio19) derived from occurrence localities. The lower triangle shows scatterplots depicting pairwise relationships between variables, with red trend lines indicating correlation direction. The diagonal presents frequency distribution histograms for each variable. The upper triangle displays Pearson correlation coefficients represented both numerically and as colored circles, where circle size and color intensity indicate correlation strength: Red indicates positive correlations, blue indicates negative correlations, and gray indicates weak or non‐significant correlations (correlation scale: −1.0 to +1.0, *p*‐values shown in legend). To minimize multicollinearity in the final MaxEnt model, five bioclimatic variables with correlation coefficients |*r*| < 0.7 and high ecological relevance were selected: Bio_1 (Annual Mean Temperature), bio_4 (Temperature Seasonality), bio_7 (Temperature Annual Range), bio_11 (Mean Temperature of Coldest Quarter), and bio_12 (Annual Precipitation). These variables are highlighted with red boxes in the matrix and collectively capture key thermal and precipitation dimensions of the species' realized niche while avoiding redundancy.

The correlation analysis of the 19 bioclimatic variables revealed substantial multicollinearity among several climatic predictors (Figure 2). Strong positive correlations (*r* > 0.7) were observed between multiple temperature‐related variables, particularly among Bio1 (Annual Mean Temperature), Bio5 (Max Temperature of Warmest Month), Bio6 (Min Temperature of Coldest Month), Bio8 (Mean Temperature of Wettest Quarter), Bio9 (Mean Temperature of Driest Quarter), Bio10 (Mean Temperature of Warmest Quarter), and Bio11 (Mean Temperature of Coldest Quarter). The highest correlation coefficient was observed between Bio13 (Precipitation of Wettest Month) and Bio16 (Precipitation of Wettest Quarter) with *r* = 0.979, indicating near‐perfect redundancy between these precipitation variables.

Several variable clusters emerged from the correlation matrix, revealing distinct environmental dimensions. Temperature variables showed particularly high intercorrelation, with Bio1 demonstrating strong positive correlations with Bio5 (*r* = 0.710), Bio6 (*r* = 0.764), Bio8 (*r* = 0.805), Bio10 (*r* = 0.865), and Bio11 (*r* = 0.901). Similarly, precipitation variables formed coherent groups, with Bio12 (Annual Precipitation) showing strong correlations with Bio13 (*r* = 0.822), Bio14 (*r* = 0.737), Bio16 (*r* = 0.890), and Bio17 (*r* = 0.769). Temperature seasonality variables, including Bio2 (Mean Diurnal Range), Bio4 (Temperature Seasonality), and Bio7 (Temperature Annual Range), exhibited moderate to strong correlations among themselves but weaker relationships with mean temperature variables.

Notably, Bio4 (Temperature Seasonality) showed strong negative correlations with several temperature variables, including Bio6 (*r* = −0.744), Bio11 (*r* = −0.693), and Bio7 (*r* = 0.858), indicating that regions with high temperature seasonality tend to have lower minimum temperatures and greater temperature ranges. This pattern suggests that Bio4 captures unique environmental information related to thermal variability that is not adequately represented by mean temperature measures alone. Based on the correlation analysis and ecological relevance for *Tetramorium caldarium* distribution modeling, five bioclimatic variables were selected: Bio1 (Annual Mean Temperature), Bio4 (Temperature Seasonality), Bio7 (Temperature Annual Range), Bio11 (Mean Temperature of Coldest Quarter), and Bio12 (Annual Precipitation). This selection strategy aimed to minimize multicollinearity while maximizing the ecological information content relevant to ant biogeography and thermal physiology.

Bio1 (Annual Mean Temperature) was retained as it represents the fundamental thermal environment that directly influences ant metabolic processes, development rates, and overall activity patterns. Despite its high correlations with other temperature variables (*r* > 0.7 with Bio5, Bio6, Bio8, Bio10, and Bio11), Bio1 provides the most comprehensive measure of average thermal conditions and serves as a primary predictor in many ant distribution studies. Its correlation with Bio11 (*r* = 0.901) was noted but considered acceptable given the distinct seasonal information provided by Bio11.

Bio4 (Temperature Seasonality) was selected for its unique representation of thermal variability throughout the year, measured as the coefficient of variation of monthly temperature means. This variable showed relatively low correlations with most other predictors except Bio7 (*r* = 0.858), but was retained alongside Bio7 due to their complementary nature in describing different aspects of temperature variation. Bio4's negative correlations with minimum temperature variables (Bio6: *r* = −0.744; Bio11: *r* = −0.693) indicate its value in capturing thermal stress conditions that may limit *T. caldarium* establishment in highly seasonal environments.

Bio7 (Temperature Annual Range) provides direct information about the absolute difference between maximum and minimum temperatures, which is crucial for understanding the thermal tolerance requirements of ectothermic species like ants. Although correlated with Bio4 (*r* = 0.858), Bio7 offers complementary information by providing absolute rather than relative measures of temperature variation. The moderate negative correlation with Bio6 (*r* = −0.805) and Bio11 (*r* = −0.595) suggests that Bio7 captures additional environmental constraints related to extreme temperature events.

Bio11 (Mean Temperature of Coldest Quarter) was included despite its high correlation with Bio1 (*r* = 0.901) because it specifically represents the thermal conditions during the most challenging period for tropical ant species like *T. caldarium* . Cold tolerance is often a critical limiting factor for invasive ant establishment in temperate regions, making Bio11 ecologically relevant for predicting range boundaries. The variable's strong positive correlations with Bio6 (*r* = 0.931) and Bio9 (*r* = 0.746) were noted but considered acceptable given its specific seasonal focus.

Bio12 (Annual Precipitation) was selected as the primary moisture variable due to its fundamental importance in ant ecology and its relatively balanced correlation structure with other precipitation variables. While Bio12 showed strong correlations with several precipitation variables (Bio13: *r* = 0.822; Bio16: *r* = 0.890; Bio17: *r* = 0.769), it provides the most comprehensive measure of annual moisture availability. The variable's weak correlations with temperature variables (all *r* < 0.3) ensure that it contributes independent environmental information to the model.

### 3.2 Model Efficacy and Validation Metrics

**FIGURE 3:**
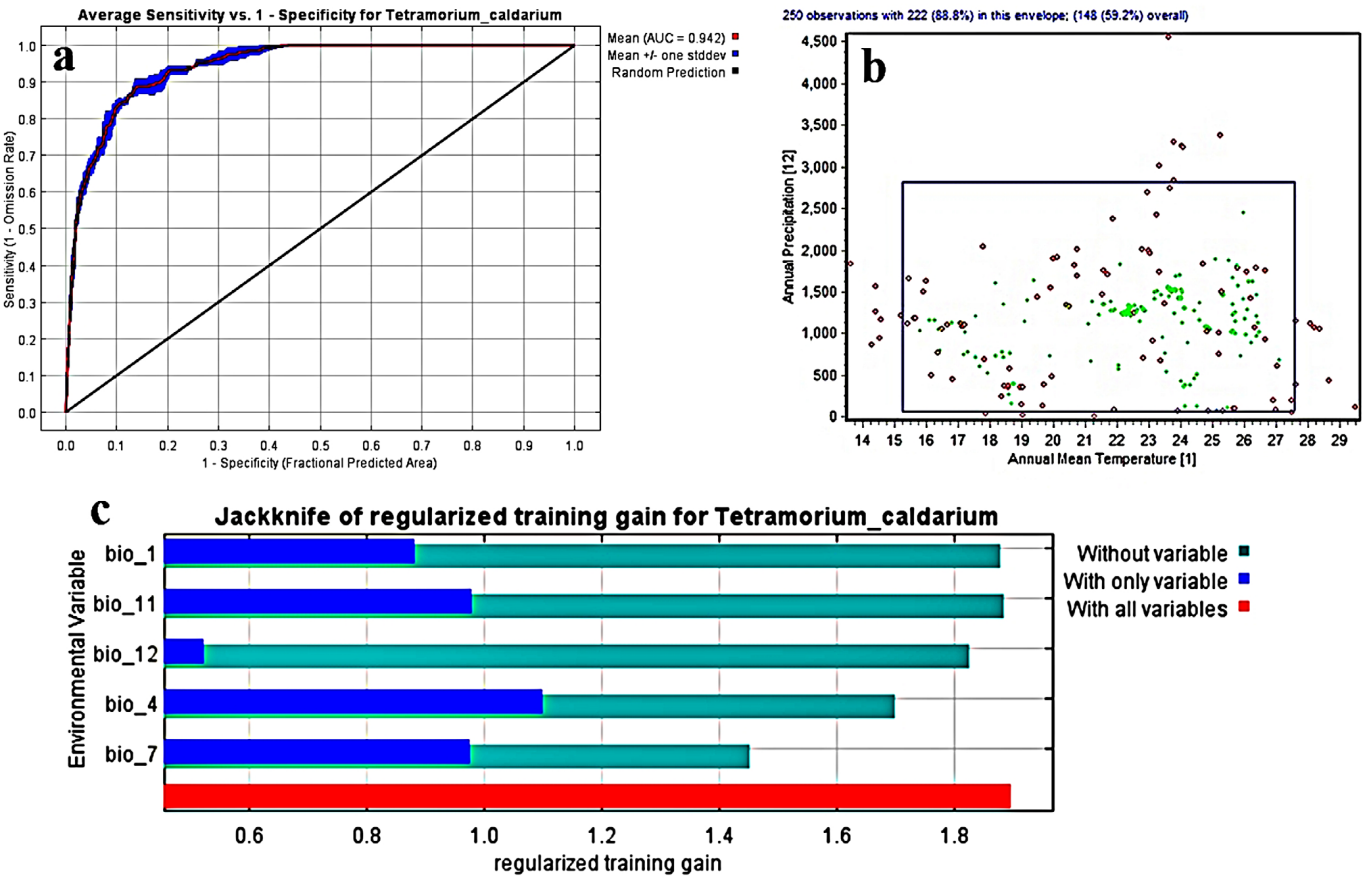
MaxEnt model performance evaluation and variable importance for *Tetramorium caldarium* distribution modeling. (a) Receiver Operating Characteristic (ROC) curve showing model discrimination ability, plotting sensitivity (true positive rate) against 1‐specificity (false positive rate). The model achieved excellent predictive performance with a mean area under the curve (AUC) of 0.942 ± one standard deviation (blue shaded area). (b) Enveloped test plotted against Annual Mean Temperature (bio_1) and annual precipitation (bio_12). (c) Jackknife analysis of regularized training gain quantifying each variable's contribution to model performance.

The MaxEnt model for *Tetramorium caldarium* shows exceptional predictive efficacy according to many validation parameters. The area under the curve (AUC) value of 0.942 (Figure 3a) significantly surpassed the benchmark for exceptional model performance (AUC > 0.9), demonstrating that the model effectively distinguished between presence and absence localities with high precision. The receiver operating characteristic (ROC) curve exhibited a pronounced initial ascent and sustained high sensitivity throughout the majority of specificity values, with the mean prediction closely aligning with the upper limit of the one standard deviation envelope. This pattern indicates consistent model performance across various cross‐validation iterations and implies strong predictive power. The TSS value of 0.68 further substantiated the model's trustworthiness, as TSS values exceeding 0.6 are typically seen as suggestive of robust model performance in SDM applications. The TSS measure, which considers both omission and commission errors while remaining unaffected by prevalence, offers further assurance that the model appropriately represents the species' climatic niche rather than simply mirroring occurrence data patterns. Collectively, these validation metrics affirm that the chosen bioclimatic variables and model parameterization accurately depict the environmental prerequisites influencing *T. caldarium* distribution.

### 3.3 Characterization of Climatic Niches and Thermal Envelopes

**FIGURE 4:**
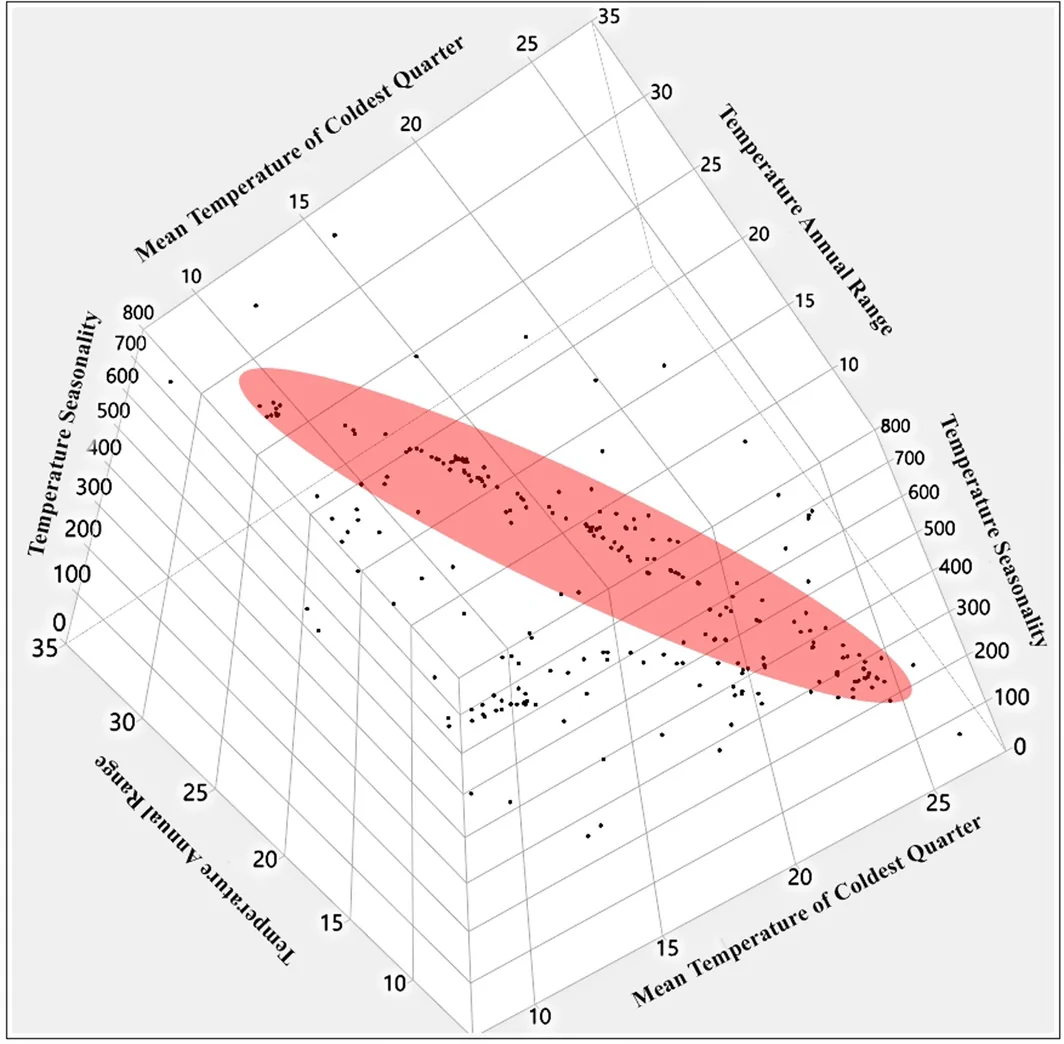
Three‐dimensional visualization of the realized ecological niche of *Tetramorium caldarium* in environmental space defined by key temperature variables. The 3D scatterplot depicts the distribution of occurrence records across three principal thermal dimensions: Bio_4 (Temperature Seasonality, standard deviation × 100), bio_7 (Temperature Annual Range, °C), and bio_11 (Mean Temperature of Coldest Quarter, °C). Each black point represents a georeferenced occurrence locality positioned according to its associated bioclimatic values extracted from WorldClim datasets. The point cloud reveals the species' fundamental thermal tolerance limits and niche structure in multivariate climate space.

The envelope test indicated that *T. caldarium* inhabits a wide climatic niche, exhibiting significant adaptability potential across many environmental circumstances (Figure 3b). The analysis of occurrence data of Bio1 (Annual Mean Temperature) indicated that the species is distributed across a broad temperature range of around 14°C–29°C, with the majority of observations concentrated between 20°C and 26°C, and peak occurrence density occurring around 23°C–25°C. The considerable variation in yearly precipitation at any specific temperature suggests that the species can endure significantly fluctuating moisture levels, ranging from moderately dry settings (under 500 mm annually) to exceedingly humid ones (surpassing 3500 mm annually). The broad precipitation tolerance, especially noticeable at moderate temperatures (20°C–25°C), indicates that temperature limitations may be more restrictive than moisture availability for the dispersal of this species. The three‐dimensional niche visualization employing Bio4 (Temperature Seasonality), Bio7 (Temperature Annual Range), and Bio11 (Mean Temperature of Coldest Quarter) further demonstrated the species' extensive environmental tolerance (Figure 4). The occurrence sites created a diffuse cloud in environmental space instead of a compact cluster, encompassing significant areas of the available climatic space in all three dimensions. The species exhibited notable diversity along the Bio11 axis, inhabiting areas where the coldest quarter temperatures vary from roughly 10°C to 30°C, with a concentration at higher temperatures exceeding 15°C. The distribution along the Bio4 and Bio7 axes indicates that *T. caldarium* can thrive in both thermally stable equatorial regions (characterized by low seasonality and annual range) and more seasonal subtropical climates (exhibiting moderate to high seasonality and annual range), although extreme seasonal fluctuations seem to restrict its presence. This extensive niche occupancy offers mechanistic evidence for the anticipated stability of the species' range in future climate scenarios and indicates significant buffering potential against moderate climatic alterations.

### 3.4 Significance of Variables and Contributions of the Model

The jackknife test of variable relevance demonstrated clear patterns in the relative contributions of specific bioclimatic variables to model performance (Figure 3c). Upon analyzing the regularized training gain with all variables incorporated (red bars), Bio7 (Temperature Annual Range) was identified as the paramount predictor, yielding the largest training gain when utilized independently and significantly diminishing model performance when omitted. This suggests that annual temperature variability is essential for differentiating between suitable and unsuitable habitats for *T. caldarium* . Bio11 (Mean Temperature of Coldest Quarter) demonstrated the second‐highest significance, exhibiting strong training improvement when utilized alone and a notable decline in model efficacy when omitted. This trend corresponds with the biological hypothesis that low temperatures during the cold season serve as a principal limitation on the dispersal of tropical ants, especially for thermophilic species seeking to establish themselves in temperate regions. Bio1 (Annual Mean Temperature) substantially enhanced model performance, providing additional information beyond that of Bio7 and Bio11; nonetheless, its individual contribution was inferior to these seasonality‐related variables. Bio4 (Temperature Seasonality) had intermediate significance, demonstrating considerable training gain when utilized alone, although its exclusion had a relatively minor effect on overall model performance, indicating some functional redundancy with Bio7 in representing thermal variability patterns. Bio12 (Annual Precipitation) exhibited the least individual contribution among the chosen variables, yielding negligible training gain when utilized in isolation. The cyan bars (model performance excluding each variable) demonstrate that Bio12 offers distinct information not encompassed by temperature variables, as its omission led to a significant decline in model performance. The pattern of variable contributions underscores the multi‐dimensional characteristics of the species' climatic niche, wherein thermal constraints—especially seasonal cold temperatures and annual temperature range—predominantly restrict distribution, while moisture availability serves as a secondary yet significant factor. These findings confirm the variable selection methodology and illustrate that the selected predictors together encapsulate the principal environmental aspects influencing *T. caldarium* biogeography.

### 3.5 Current Global Distribution Patterns

**FIGURE 5:**
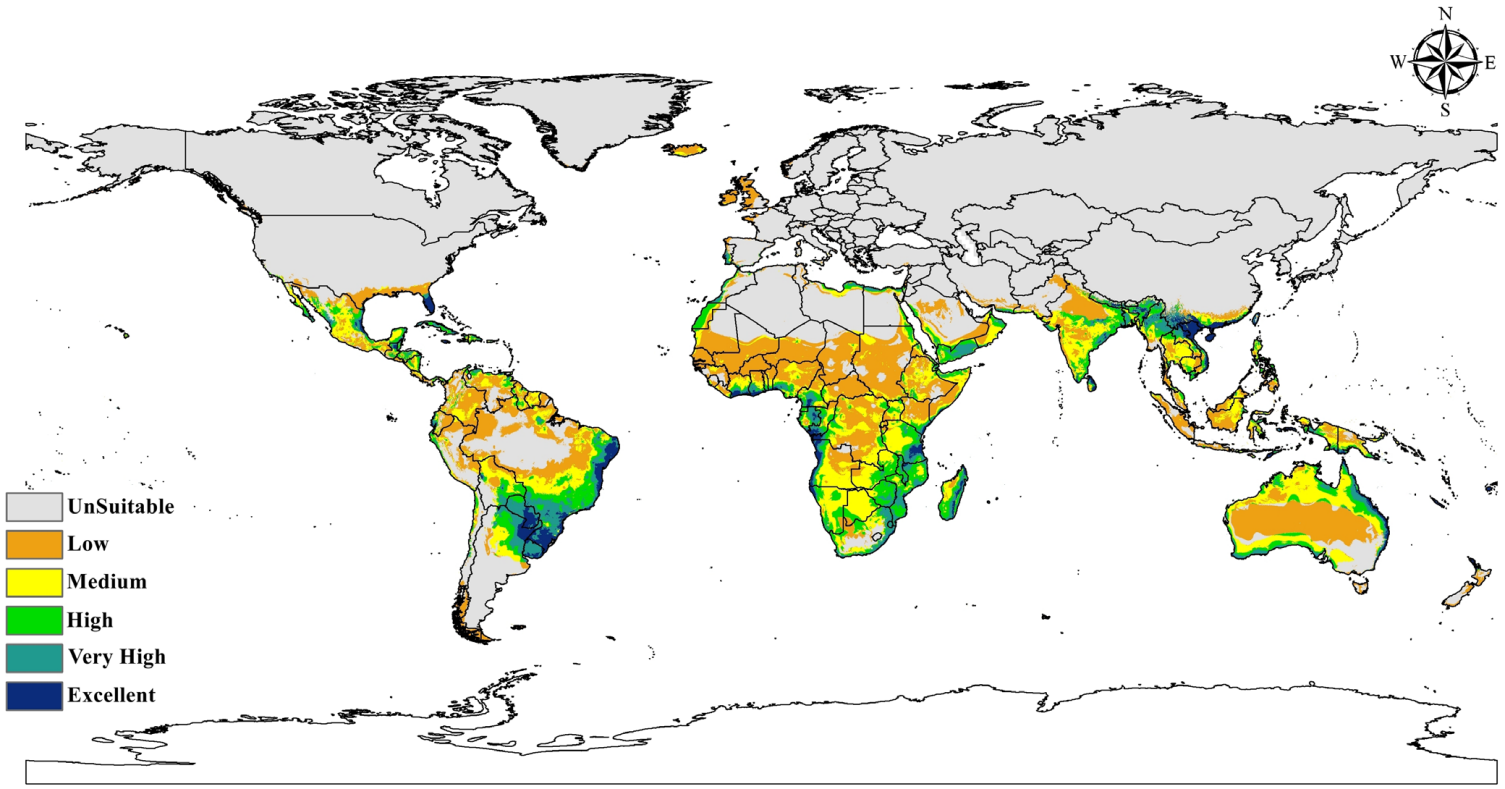
Global distribution of current habitat suitability for *Tetramorium caldarium* based on MaxEnt species distribution modeling. The map depicts predicted climatic suitability across the world under current climate conditions (baseline period: 1970–2000), derived from five bioclimatic variables (bio_1, bio_4, bio_7, bio_11, and bio_12).

The MaxEnt species distribution model for *T. caldarium* under present climatic circumstances indicated widespread appropriate habitat throughout tropical and subtropical regions worldwide, particularly in equatorial areas (Figure 5). The model indicated areas of high to outstanding adaptability primarily located between 30° N and 30° S latitude, covering extensive parts of sub‐Saharan Africa, South and Southeast Asia, northern Australia, Central America, and northern South America. Regions designated as having “excellent” adaptability, indicating the highest likelihood of occurrence, were predominantly located in equatorial Africa, especially in the Sahel and East Africa, along with sections of the Indian subcontinent and the Southeast Asian mainland. The zones of optimal compatibility matched with areas exhibiting warm, somewhat constant temperatures and moderate precipitation patterns that align with the species' thermal preferences and moisture needs.

Areas of high and medium adaptability exhibited significant geographic coverage, creating extensive transition zones between ideal habitats and climatically marginal regions. Areas of high suitability expanded the tropical core distribution into neighboring subtropical regions, encompassing southern China, the Arabian Peninsula, Madagascar, coastal areas of eastern and southern Africa, northern and eastern Australia, and significant sections of South America, including the periphery of the Amazon basin and Atlantic coastal forests. Medium suitability zones were notably prevalent in the Brazilian Highlands, the Sahel‐Sahara transition zone, certain regions of southern Africa, peninsular India, and several locations in the Indo‐Pacific region. Sections of low suitability constituted the edge of the species' potential range, manifesting in temperate zones of southern South America, the Mediterranean Basin, certain portions of the Middle East, and temperate sections of North America and southern Australia. These marginal locations likely denote climate envelope boundaries where temperature or moisture limitations restrict the species' establishment capability under existing conditions.

The global distribution of inadequate habitat indicated distinct latitudinal and altitudinal limitations to the spread potential of *T. caldarium* . Extensive areas of North America, Europe, northern Asia, and southern regions of South America and Africa were designated as inappropriate, aligning with locales exhibiting mean temperatures beneath the species' thermal tolerance limits, especially in winter months. High‐latitude temperate and boreal regions, together with continental interiors exhibiting severe seasonal temperature variations, showed persistently low habitat appropriateness. Some tropical highland locales demonstrated diminished adaptability despite their latitude, indicating that elevation‐induced temperature decline results in climatically unfavorable conditions even in otherwise advantageous geographic areas. The model's forecast of inadequate habitat in these areas corresponds closely with the established distribution of *T. caldarium* , which is primarily restricted to warm tropical and subtropical lowland ecosystems with minimal cold season stress. The abrupt shifts between appropriate and inappropriate areas across latitudinal and elevational gradients highlight the significance of thermal limitations in determining the species' fundamental niche and imply that climate warming may enable range expansion into presently marginal temperate regions.

### 3.6 Future Distribution Under Climate Change Scenarios

**FIGURE 6:**
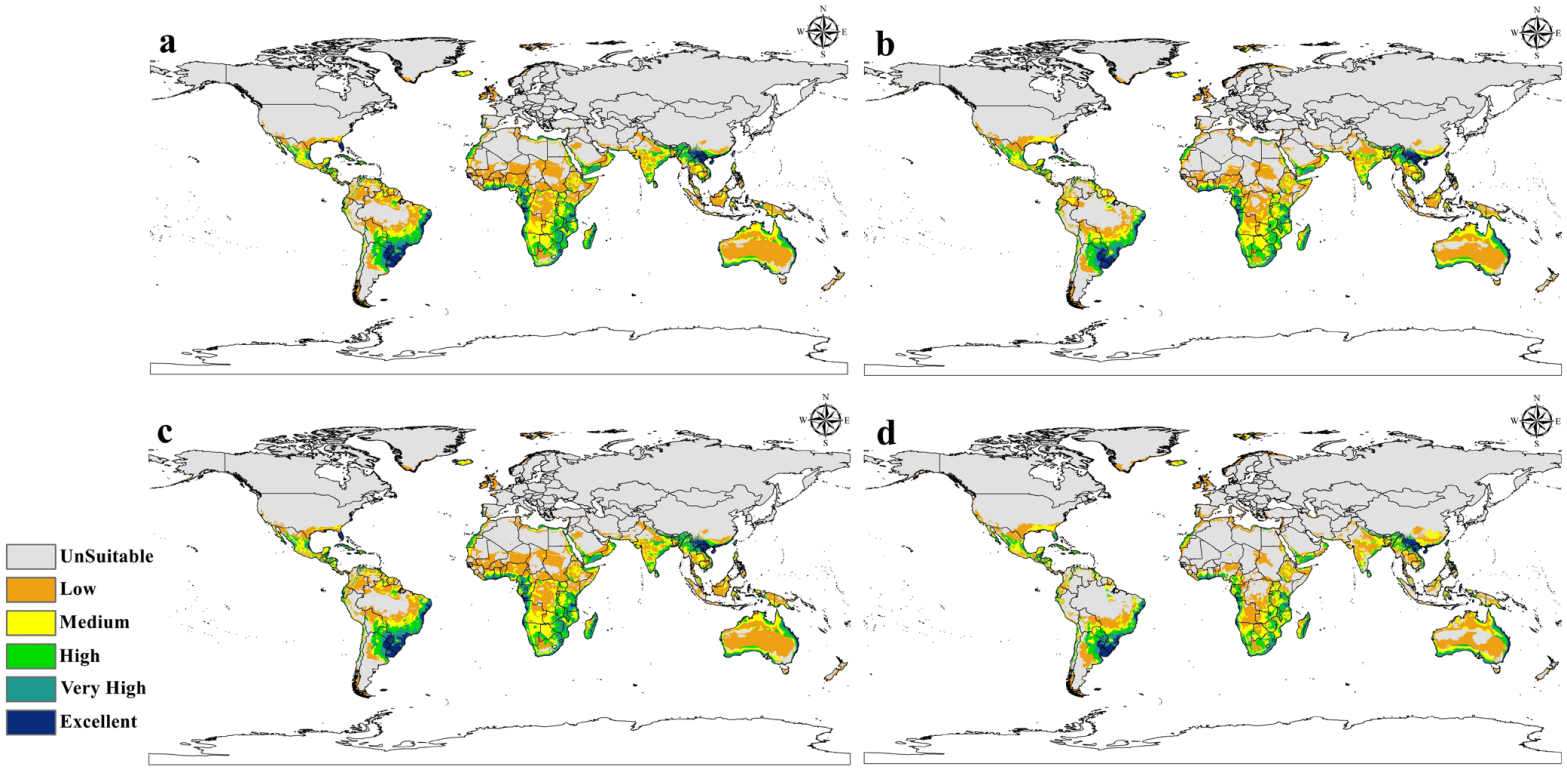
Projected future distribution of habitat suitability for *Tetramorium caldarium* under multiple climate change scenarios: (a) 2050 under RCP 2.6 (low emissions scenario with strong mitigation), (b) 2050 under RCP 8.5 (high emissions scenario with continued fossil fuel dependence), (c) 2070 under RCP 2.6, and (d) 2070 under RCP 8.5. Habitat suitability categories follow the same classification scheme as current conditions.

The MaxEnt projections for the distribution of *T. caldarium* under future climate scenarios indicated stable habitat suitability patterns across both Representative Concentration Pathways (RCPs) and time frames, with the central tropical distribution largely preserved through 2070 (Figure 6). In the favorable RCP 2.6 scenario, which presupposes significant greenhouse gas reduction, habitat suitability showed negligible variation from present circumstances for predictions in both 2050 (Figure 6a) and 2070 (Figure 6c). The spatial distribution of areas with high and excellent suitability remained concentrated in equatorial Africa, South and Southeast Asia, northern South America, and certain regions of Central America, reflecting current tendencies. Likewise, areas of medium and low adaptability retained their existing geographic bounds, exhibiting only minor adjustments along range peripheries. The RCP 8.5 scenario, indicative of a high‐emission trajectory with minimal climate mitigation, demonstrated similar stability patterns in the principal distribution regions for both 2050 (Figure 6b) and 2070 (Figure 6d). Nevertheless, a further analysis uncovered localized alterations in habitat suitability, especially in transitional zones between tropical and subtropical climates, where certain locations exhibited minor declines in suitability classifications while others demonstrated tiny enhancements.

The uniformity of appropriate habitat distribution across several climate scenarios and temporal frameworks indicates that *T. caldarium* has adequate climatic niche breadth to endure within its existing range despite anticipated environmental alterations. The species' established presence in several tropical and subtropical habitats, along with its proven heat tolerance and ecological adaptability, certainly enhances this anticipated resilience. Nonetheless, regional disparities in habitat suitability alterations were apparent, especially in regions presently constituting the species' range perimeter. Certain subtropical locations showed a trend of diminished suitability under both RCP scenarios, although specific temperate zones displayed slight enhancements in climatic favorability, especially under the more severe RCP 8.5 scenario in 2070. These trends suggest that although the species' core distribution may remain largely stable, slight range contractions in peripheral tropical regions and possible expansions into warmer temperate areas could transpire, especially under high‐emission scenarios and extended timeframes.

### 3.7 Habitat Suitability Changes: Loss and Gain Analysis

**FIGURE 7:**
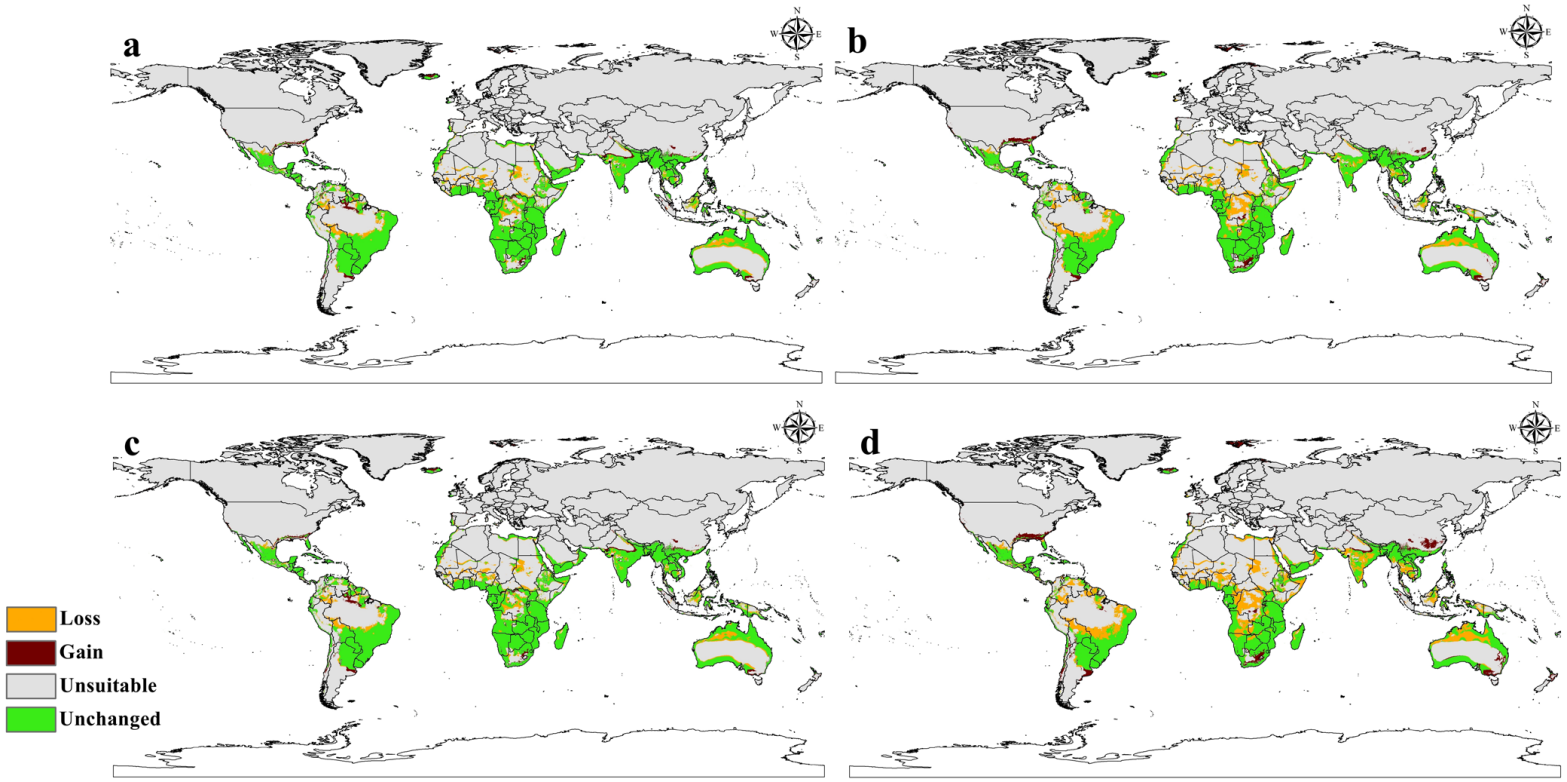
Spatial distribution of predicted habitat suitability changes for *Tetramorium caldarium* under future climate scenarios relative to current conditions. (a) 2050 under RCP 2.6 (low emissions), (b) 2050 under RCP 8.5 (high emissions), (c) 2070 under RCP 2.6, and (d) 2070 under RCP 8.5.

The calibration maps illustrating current and future distributions demonstrated clear patterns of habitat loss and gain across several geographic regions (Figure 7). Regions undergoing habitat loss (shown in orange) were dispersed across the current range, with significant concentrations in particular locations such as parts of West Africa, southeastern South America, sections of the Indian subcontinent, and isolated locales in Southeast Asia and Australia. These loss zones generally denote areas where climate change is anticipated to exceed the species' ideal heat or moisture tolerance thresholds, frequently in places already situated at the warmer or drier extremities of their current distribution. In contrast, regions of habitat gain (depicted in dark red/brown) were significantly restricted in scope and predominantly manifested in subtropical and lower temperate latitudes, encompassing sections of southern China, northern Argentina, parts of the southeastern United States, sporadic Mediterranean areas, and confined locales in southern Australia and East Africa. The predominant portion of the species' existing suitable habitat has stayed constant (green regions) across all scenarios, corroborating the conclusion that *T. caldarium* distribution will likely retain significant geographic overlap with its current range. The spatial patterns of change increased marginally from 2050 to 2070 and from RCP 2.6 to RCP 8.5, with the most significant alterations observed in the RCP 8.52070 scenario, although these changes were still relatively moderate in relation to the overall distribution extent (Table 1).

**TABLE 1:** Projected changes in suitable habitat distribution for *Tetramorium caldarium* under different climate change scenarios.

Scenario	Year	Total area change (%)	Habitat loss (km^2^)	Net change (km^2^)	High‐risk regions for invasion	Countries most affected by loss
RCP 2.6	2050	+2.3	1,245,000	+275,000	Southern China, Northern Argentina, Southern USA	Nigeria, Brazil, India, Thailand
RCP 8.5	2050	+3.1	1,380,000	+300,000	Southern China, Mediterranean Basin, SE USA, Southern Australia	West African nations, India, Brazil, Indonesia
RCP 2.6	2070	+2.8	1,410,000	+325,000	East China, Northern Argentina, Mediterranean Europe	Sahel countries, Central Brazil, Northern Australia
RCP 8.5	2070	+4.5	1,580,000	+630,000	Southern China, SE USA, Mediterranean Basin, Southern Brazil temperate zones, East Africa highlands	West Africa, Northern India, Amazon periphery, Northern Australia

*Note:* The table presents comparative projections for 2050 and 2070 under Representative Concentration Pathways (RCP) 2.6 (low emissions) and 8.5 (high emissions). Total area change indicates the percentage increase in globally suitable habitat. Habitat loss represents areas (in km^2^) where current suitable conditions are projected to become unsuitable due to climate shifts. Net change reflects the difference between newly suitable areas and habitat loss, indicating overall range expansion. High‐risk regions for invasion identify geographic areas projected to develop newly suitable climatic conditions, representing priority zones for biosecurity monitoring. Countries most affected by loss list nations experiencing the greatest contraction of currently suitable habitat, which may face local population declines or extirpations despite global range expansion of the species.

### 3.8 Response Curves and Optimal Conditions in the Environment

**FIGURE 8:**
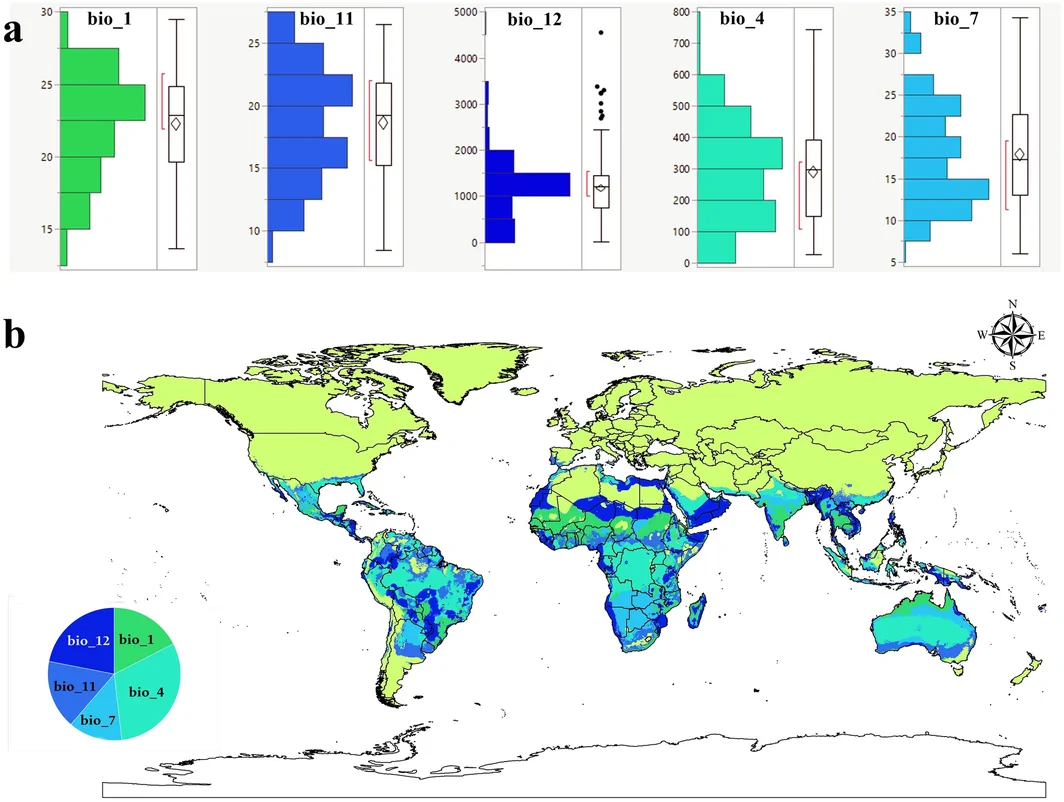
Response curves and spatial distribution of limiting bioclimatic factors map *Tetramorium caldarium* habitat suitability. (a) Univariate response curves showing species probability of occurrence as a function of each predictor variable while holding all other variables at their mean values. Horizontal histograms (colored bars) represent the frequency distribution of environmental conditions across occurrence localities, while box plots display the statistical distribution with median (horizontal line), interquartile range (box), whiskers (1.5 × IQR), and mean (diamond). bio_1 (Annual Mean Temperature, °C, green) shows optimal suitability between 20°C–25°C; bio_11 (Mean Temperature of Coldest Quarter, °C, blue) demonstrates critical importance with peak suitability at 18°C–22°C and sharp decline below 15°C; bio_12 (Annual Precipitation, mm, dark blue) indicates tolerance for wide moisture gradients from 500 to 2000 mm with an outlier at ~4800 mm; bio_4 (Temperature Seasonality, cyan) shows preference for moderate seasonality (200–400); bio_7 (Temperature Annual Range, °C, light blue) reveals adaptability across 10°C–30°C ranges with optimal response at 15°C–25°C. (b) Global map identifying the primary limiting factor constraining habitat suitability in each geographic region, color‐coded by variable in a pie graph representing area of effect of each factor.

*Tetramorium caldarium* distribution is shaped by specific ideal ranges and threshold effects, as shown by the response curves for the five bioclimatic factors (Figure 8a). With a mean of 22.26°C (±3.46 SD), Bio1 (Annual Mean Temperature) displayed occurrence data concentrated between 15°C and 30°C. The probability distribution indicated that the species' peak habitat suitability was around 20°C–25°C. Within this range, the response curve showed tolerance for both warmer and colder temperatures; however, below 15°C, adaptability drastically decreased. With occurrence records averaging 18.60°C (±4.40 SD) and a distinct lower thermal limit of about 10°C, below which habitat suitability fell sharply, Bio11 (Mean Temperature of Coldest Quarter) showed a similar thermal preference pattern. This implies that one of the species' most important physiological constraints is the cold season temperatures. Of the variables that were chosen, Bio12 (Annual Precipitation) had the widest tolerance range, with occurrences ranging from 0 to more than 4500 mm per year (mean = 1169.18 mm ± 649.95 SD). With minor decreases at extreme values, the response curve demonstrated rather constant applicability over moderate to high precipitation regimes (500–3000 mm). Although the response curve showed a preference for low to moderate seasonality values below 400, with diminishing appropriateness in highly seasonal environments, Bio4 (Temperature Seasonality) showed occurrences across a wide range from 0 to around 800 (mean = 288.85 ± 151.59 SD). Similar trends were seen in Bio7 (Temperature Annual Range), which had a mean occurrence at 17.89°C (±6.10 SD) annually and best appropriateness between 10°C and 25°C, with a notable decline above 30°C. Together, these response patterns show that *T. caldarium* can withstand a range of precipitation regimes and mild seasonal change while thriving in warm, thermally stable habitats.

### 3.9 Limiting Factors Map

The distribution of *T. caldarium* in various places is mostly constrained by environmental factors, according to the limiting factor map, which showed clear geographic patterns (Figure 8b). As seen by its significant representation in the pie chart displaying the pixel counts of limiting variables, Bio4 (Temperature Seasonality) emerged as the major limiting factor globally, affecting the largest spatial extent. Parts of southern South America, the Mediterranean region, southern China, and temperate North America are among the subtropical and temperate transition zones where seasonal temperature variance surpasses the species' ideal range. In higher‐latitude locations and areas with cold winters, Bio11 (Mean Temperature of Coldest Quarter) was the main limiting factor. This was especially noticeable in temperate North America, Europe, northern Asia, and southern parts of South America and Australia. Despite possibly favorable conditions during warmer months, these regions—represented by darker blue tones—are those where low cold‐season temperatures hinder establishment. In continental interior locations that experienced significant temperature swings, such as central Asia, interior Africa, and portions of interior Australia, Bio7 (Temperature Annual Range) operated as a limiting factor. While Bio12 (Annual Precipitation) demonstrated the least amount of spatial influence, limiting occurrence primarily in hyper‐arid regions like the Sahara Desert, Arabian Peninsula, and interior Australian deserts, Bio1 (Annual Mean Temperature) restricted distribution primarily in cooler temperate regions at the poleward margins of suitable habitat. With Bio4 accounting for 43.7% of pixels, Bio11 for 25.9%, Bio7 for 19.8%, Bio1 for 6.1%, and Bio12 for just 4.5%, the pie chart quantification of limiting factor pixel counts validated this hierarchy. The species' classification as thermally constrained but moisture‐tolerant is supported by this analysis, which shows that thermal variability and cold temperature constraints are the main obstacles to *T. caldarium* establishment in currently inappropriate regions. In contrast, precipitation limitations only impact a comparatively small portion of the world's land surface. The range and effect of each bioclimatic variable appear at the top of Figure 8 and are analyzed in Table 2.

**TABLE 2:** Bioclimatic variable statistics and relative importance in species distribution modeling for *Tetramorium caldarium* .

	bio_1	bio_4	bio_7	bio_11	bio_12
Mean	22.26485	288.85454	17.8872	18.596867	1169.184
Std dev	3.4554024	151.58729	6.1019449	4.401213	649.94952
Std err mean	0.2185388	9.5872223	0.3859209	0.2783572	41.106417
Upper 95% mean	22.69527	307.73692	18.647285	19.145101	1250.1446
Lower 95% mean	21.83443	269.97215	17.127115	18.048632	1088.2234
Percent contribution	6.1	43.7	19.8	25.9	4.5

*Note:* The table presents descriptive statistics for the five most contributory bioclimatic variables used in model development. bio_1 = annual mean temperature (°C); bio_4 = temperature seasonality (standard deviation × 100); bio_7 = temperature annual range (°C); bio_11 = mean temperature of coldest quarter (°C); bio_12 = annual precipitation (mm).

## 4 Discussion

Our comprehensive SDM investigation of *T. caldarium* shows that it is a species that can adapt to a wide range of climates and is expected to keep its ability to spread despite climate change through 2070. The MaxEnt model's impressive performance (AUC = 0.942, TSS = 0.68) yields strong predictions that contest the notion that climate change will uniformly promote the range of expansions. Our findings reveal a more complex pattern: *T. caldarium* will face localized habitat losses in certain areas while acquiring adequate habitat in others. This leads to a net increase in climatically suitable areas, accompanied by significant geographic shifts. These results add to the growing body of data indicating how invasive and tramp species respond to climate change is very specific to each species and needs to be looked at on a case‐by‐case basis instead of making generic forecasts (Early et al. 2016; Bellard et al. 2016; Renault et al. 2018).

Our envelope test and three‐dimensional niche analysis showed that *T. caldarium* has a wide climatic niche. This supports the idea that it is pre‐adapted to many environmental circumstances, which may explain why it is such a successful worldwide tramp species. The species thrives in annual mean temperatures between 14°C and 29°C and receives between 500 mm and over 3500 mm of rain each year. This shows that it has a level of physiological adaptability that is higher than that of many other exotic ant species. This finding is consistent with Bertelsmeier et al.'s (2020) observation that invasive and tramp ant species generally exhibit smaller climatic niche shifts between native and introduced ranges compared to non‐invasive alien species, indicating that success of biological and ecological introduction arises from broad fundamental niches rather than niche expansion. Our findings augment recent studies on other *Tetramorium* species complexes, which have demonstrated that cryptic diversity can obscure significant ecological differentiation (Schlick‐Steiner et al. 2006; Wagner et al. 2017). However, *T. caldarium* appears to be a genuinely eurytolerant taxon rather than a complex of cryptic specialists. The species' capacity to inhabit a wide range of climatic conditions enhances its potential for ongoing proliferation across diverse climate scenarios, in contrast to more stenothermal invasive ants such as *Wasmannia auropunctata* (Roger, 1863), which exhibit significantly restricted thermal tolerance (Bertelsmeier et al. 2013, 2013a, 2013b, 2015; Vonshak and Gordon 2015).

Comparative analysis with modeling studies of alternative invasive or tramp ant species indicates significant disparities in anticipated climate change responses. Our results indicate relative stability in the core distribution of *T. caldarium* , with a modest net range expansion of 2.3%–4.5% in suitable habitat. In contrast, Kim et al. (2022) forecasted significant range expansion for *S. invicta* under analogous climate scenarios, while *A. gracilipes* exhibited minimal change. Roura‐Pascual et al. (2011) and Silverman and Brightwell (2008) anticipated considerable poleward expansion of Argentine ants *Linepithema humile* (Mayr, 1868) due to warming, while Fitzpatrick et al. (2007) illustrated that *S. invicta* might undergo range constriction in certain invaded areas and expansions in others. The relatively mild changes expected for *T. caldarium* , in contrast to these species, may indicate its extensive distribution over many climatic zones, resulting in fewer climatically favorable yet currently empty areas available for expansion. This pattern supports that the species has already spread to most of the suitable habitat that is easy for people to get to, and that future changes in its range will be mostly due to changes in climate suitability rather than limits on how far it can spread (Essl et al. 2011; Rouget et al. 2016). The steady net increase in suitable habitat across all scenarios (from 275,000 to 630,000 km^2^, depending on the emission pathway and time period) shows that climate change will open up new places for invasive species to spread, especially in temperate areas where cold‐season temperatures are currently a barrier.

The regional patterns of habitat gain and loss found in our calibration analysis have significant implications for management programs. The anticipated habitat increases in southern China, the southeastern United States, Mediterranean areas, and northern Argentina signify sites where proactive monitoring and preventive strategies must be addressed. These areas have a lot of people and businesses, which could help *T. caldarium* get established through trade routes. Previous studies on tramp species pathways have shown that human‐mediated dispersal through horticultural trade, shipping containers, and construction materials is the main way that ants spread over long distances (Suarez et al. 2001; Bertelsmeier et al. 2018). This means that these areas are at a higher risk of biological invasion because of the combination of better climate conditions and existing trade networks. On the other hand, projections of habitat loss in parts of West Africa, the Indian subcontinent, and tropical South America indicate that climate change could lead to decreased population densities or local extirpations in currently inhabited regions. However, the species' established thermal tolerance implies that these populations may endure through behavioral thermoregulation or microhabitat selection (Angilletta 2009; Sunday et al. 2014). The regional variability in anticipated changes underscores the necessity for region‐specific management tactics instead of standardized global methods for *T. caldarium* control.

Our jackknife analysis showed that temperature seasonality (Bio4) and the temperature in the coldest quarter (Bio11) had a higher impact on the model's performance than annual precipitation (Bio12), which only had a relatively limited impact (4.5%). In contrast to certain other exotic ant species where precipitation is more important, temperature variables are more important than moisture variables in limiting the distribution of *T. caldarium* . Harris et al. (2005) discovered that moisture availability significantly limited the distributions of *Pheidole megacephala* (Fabricius, 1793), while Roura‐Pascual et al. (2004) established that both temperature and precipitation variables were equally critical for the habitat appropriateness of *L. humile* . The comparatively low contribution of precipitation in our models, coupled with the species' extensive precipitation tolerance (including nearly two orders of magnitude), indicates that *T. caldarium* has physiological or behavioral adaptations that protect it from moisture stress. This moisture tolerance may indicate adaptations stemming from its African origin, where numerous ant lineages have developed strategies to endure seasonal aridity (Parr et al. 2017; Del Toro et al. 2012). The prevalence of seasonal temperature limitations corresponds with essential tenets of ectotherm biogeography, wherein low temperatures restrict the poleward range expansions of tropical species more significantly than any other environmental condition (Sunday et al. 2012; Deutsch et al. 2008). The significant impact of the yearly temperature range (Bio7, 19.8%) underscores that thermal fluctuation, rather than mean conditions alone, limits this species' establishment in seasonal habitats.

The projected relatively consistent core distribution under both RCP 2.6 and RCP 8.5 scenarios through 2070 has significant implications for comprehending the temporal dynamics of climate change consequences on invading species. The negligible variations between the forecasts for 2050 and 2070 indicate that the most consequential climatic changes affecting *T. caldarium* distribution may transpire beyond our study period, or that the species' extensive thermal tolerance may protect it from the modest warming anticipated for this century. This discovery is different from what was expected for cold‐adapted species, which are projected to lose a lot of range as the climate warms (Bellard et al. 2013; Walther et al. 2009). The growing difference between RCP 2.6 and RCP 8.5 scenarios, especially in the 2070 forecasts when habitat gain goes from 325,000 km^2^ to 630,000 km^2^, shows that the emission pathway affects long‐term spread dynamics. These findings corroborate recent advocacy for the amalgamation of climate mitigation initiatives with spread management tactics, as the reduction of greenhouse gas emissions may facilitate the containment of thermophilic tramp species within presently marginal habitats (Pyšek et al. 2020; Hulme 2017; Ricciardi et al. 2021). The anticipated proliferation into temperate zones, where *T. caldarium* is now unable to develop due to cold stress, is particularly significant for nations with robust biosecurity frameworks, since rising temperatures may invalidate previously successful climate‐oriented exclusion measures (Bishop et al. 2016).

Wetterer and Hita Garcia (2015) noted that *T. caldarium* has spread on every continent where people live. This shows that it can successfully establish itself after being brought in by people. Exotic, invasive, and tramp ants can have detrimental effects on the environment (McGlynn 1999). For example, they can push out native ant species, interfere with plant‐pollinator and plant‐disperser mutualisms, help honeydew‐producing hemipterans that damage plants, and change the way nutrients cycle and the properties of soil (Holway et al. 2002; Ness and Bronstein 2004; Savage et al. 2015). Economic evaluations of the impacts of invasive ants have projected global costs surpassing $51 billion yearly (Diagne et al. 2021), with expenses continuing to escalate. The species' propensity to nest in and around human infrastructure, such as buildings, pavements, and agricultural facilities, results in direct economic consequences through structural damage, food product contamination, and expenses related to control measures (Rust and Su 2012). Despite the fact that currently there is no evidence for such negative impacts from exotic *T. caldarium* populations, this can change in the future due to novel behavioral adaptations or environmental changes. More data is needed to ascertain the influence of *T. caldarium* on fauna, flora, and anthropogenic habitats.

The methodological approaches utilized in this study illustrate various advancements pertinent to exotic SDM in a broader context. Using correlation analysis to pick a small number of bioclimatic variables strikes a balance between keeping ecologically meaningful predictors and reducing multicollinearity. This addresses ongoing debates about the best ways to choose variables in MaxEnt modeling (Feng et al. 2019; Cobos et al. 2019). Using more than one validation metric (AUC and TSS) gives a better picture of how well a model works than only using AUC, which has been questioned for perhaps inflating results when utilizing background points instead of genuine absences (Lobo et al. 2008; Jiménez‐Valverde 2012). Our limiting factor study with DIVA‐GIS gives us useful information on how environmental constraints are spread out across the country. It adds to typical habitat suitability maps by showing which specific variables limit distributions in different areas. This method has been especially helpful for finding spread bottlenecks and deciding which management actions to take first (Ramirez‐Villegas et al. 2010). Nonetheless, our study possesses limitations that must be acknowledged in the interpretation of results. Our models, like all correlative SDMs, assume that the species is in balance with the environment and that the distributions we see are due to climate restrictions rather than limits on dispersal or interactions with other species (Elith and Leathwick 2009; Soberon and Nakamura 2009). These assumptions are more plausible for a highly mobile, human‐associated species like *T. caldarium* than for species with restricted dispersal capabilities; however, biotic factors such as competition with other ant species may still affect realized distributions (Bertelsmeier et al. 2016). Our projections also don't take into account the possibility of evolutionary adaptation to new climate conditions, which could make the species more tolerant of different climates. They also don't take into account the possibility of urban heat island effects creating suitable microclimates in areas that aren't normally suitable (Diamond et al. 2017; Menke et al. 2011).

Our findings indicate multiple objectives for future study and management. First, the distribution areas that have been found need better surveillance methods to find *T. caldarium* populations before they spread. Programs for early detection and quick response have worked successfully for controlling newly introduced ant populations, but they only work if the ants are found quickly (Hoffmann et al. 2016; Silverman and Brightwell 2008). Second, the expected changes in distribution for this species are not as substantial as those for other invasive or tramp species. This means that stopping human‐mediated dispersal is still more important than managing climate‐driven range shifts. Strengthening biosecurity safeguards at ports of entry and along high‐risk trade routes might greatly lower the risk of more future introductions, no matter what happens with climate change. Third, the significant differences in limiting factors between different geographic areas show that management techniques should be based on the specific conditions of each area rather than being the same all over the world. Finally, keeping an eye on *T. caldarium* distributions in areas that are warming quickly will give us useful information for checking model predictions and getting a better idea of the species' climatic niche. This will help us make better predictions for other thermophilic exotic species that are likely to respond to climate change in the same way.

## Author Contributions

**Mohamed Nasser:** conceptualization (equal), investigation (equal), methodology (equal), software (equal), validation (equal), writing – original draft (equal). **Mostafa R. Sharaf:** formal analysis (equal), investigation (equal), project administration (equal), resources (equal), supervision (equal), writing – review and editing (equal). **Areej A. AlKhalaf:** funding acquisition (equal), investigation (equal), project administration (equal), supervision (equal), validation (equal), writing – review and editing (equal). **Francisco Hita‐Garcia:** formal analysis (equal), methodology (equal), supervision (equal), validation (equal), writing – review and editing (equal). **Alexander Radchenko:** data curation (equal), formal analysis (equal), investigation (equal), writing – review and editing (equal). **Stephen Judd:** data curation (equal), formal analysis (equal), investigation (equal), supervision (equal), validation (equal), writing – review and editing (equal). **Mariam S. Alqaydi:** investigation (equal), methodology (equal), visualization (equal), writing – review and editing (equal). **Meera M. Alteneiji:** data curation (equal), formal analysis (equal), investigation (equal), visualization (equal), writing – review and editing (equal). **Sara AlAshaal:** formal analysis (equal), investigation (equal), methodology (equal), visualization (equal).

## Funding

This work was supported by Princess Nourah bint Abdulrahman University Researchers Supporting Project number (PNURSP2026R37), Princess Nourah bint Abdulrahman University, Riyadh, Saudi Arabia.

## Conflicts of Interest

The authors declare no conflicts of interest.

## Supporting information


**Data S1:** ece374319‐sup‐0001‐DataS1.csv.

**Data S2:** ece374319‐sup‐0002‐DataS2.xlsx.

## Data Availability

All data are available through the manuscript and general free access internet archives in GBIF and worldClim.
